# Comparative Analysis of the Effects of Upconversion Nanoparticles on Normal and Tumor Brain Cells

**DOI:** 10.32607/actanaturae.11033

**Published:** 2020

**Authors:** T. A. Mishchenko, E. V. Mitroshina, A. S. Smyshlyaeva, E. L. Guryev, M. V. Vedunova

**Affiliations:** National Research Lobachevsky State University of Nizhny Novgorod, Nizhny Novgorod, 603950 Russia; Privolzhsky Research Medical University, Nizhny Novgorod, 603005 Russia

**Keywords:** upconversion nanoparticles, primary hippocampal cultures, glioma, U-251 MG, GL261, toxicity, functional neural network activity

## Abstract

Glioma is the most aggressive type of brain tumors encountered in medical practice. The high frequency of diagnosed cases and risk of metastasis, the low efficiency of traditional therapy, and the usually unfavorable prognosis for patients dictate the need to develop alternative or combined approaches for an early diagnosis and treatment of this pathology. High expectations are placed on the use of upconversion nanoparticles (UCNPs). In this study, we have produced and characterized UCNPs doped with the rare-earth elements ytterbium and thulium. Our UCNPs had photoluminescence emission maxima in the visible and infrared spectral regions, which allow for deep optical imaging of tumor cells in the brain. Moreover, we evaluated the toxicity effects of our UCNPs on a normal brain and glioma cells. It was revealed that our UCNPs are non-toxic to glioma cells but have a moderate cytotoxic effect on primary neuronal cultures at high concentrations, a condition that is characterized by a decreased cellular viability and changes in the functional metabolic activity of neuron-glial networks. Despite the great potential associated with the use of these UCNPs as fluorescent markers, there is a need for further studies on the rate of the UCNPs accumulation and excretion in normal and tumor brain cells, and the use of their surface modifications in order to reduce their cytotoxic effects.

## INTRODUCTION

For many years, neurooncology has been one of the acute problems in public health. According to the International Agency for Research on Cancer, the incidence of diagnosed cases of brain tumors stood at 6–19 cases for men and 4–18 cases for women per 100,000 of the population in 2016–2017 and shows a steady tendency toward increase. Gliomas are the most common and aggressive types of brain tumors, which are characterized by short survival time (≤ 5 years), as well as a high incidence of metastasis and death rate [1]. The optimal set of procedures currently being used in clinical practice includes microsurgery, chemotherapy, and radiotherapy [2, 3]. However, these treatment methods neither significantly increase patient survival rate nor improve his/her quality of life.

In this field, high expectations are placed on the use of nanomaterials. Thanks to their small size, variety of conformations, and chemical structure of their core and shell, nanoparticles can be used in a variety of cases. For instance, nanoparticles can act as a carrier for the targeted delivery of a drug to the tumor site. The nanoscale delivery system can penetrate the blood–brain barrier (BBB), protect the chemotherapeutic agent from early cleavage, improve its pharmacokinetics, as well as reduce the dose of the active substance, thus minimizing the risk of non-specific toxicity [4]. A number of experimental studies have shown that the use of nanoparticles loaded with doxorubicin [5], paclitaxel [6], as well as the combination of paclitaxel and angiopep (a drug facilitating penetration of the tumor vessels through the BBB) [7], in the treatment of gliomas provides specificity to the accumulation of the chemotherapeutic agent in the target cells, the inhibition of tumor cell growth and proliferation, and their death. There are also studies related to the development of carrier nanoparticles capable of inducing immunogenic cell death. Formation of a stable adaptive T cell immune response allows for effective elimination of infiltrative tumor cells, which can become one of the causes of metastases [8].

Nanoparticles can also act as fluorescent markers for the visualization of tumor cells to determine clear boundaries for resection during surgery. Of particular interest are upconversion nanoparticles (UCNPs). In addition to the fact that UCNPs can be used as drug carriers [9], they are photoluminescent (PL) nanoparticles capable of converting low-energy photons to higher energy photons. UCNPs possess unique photophysical properties and photochemical stability, which allow for deep bioimaging [10-13]. UCNPs are excited by low-intensity infrared (IR) light (1–103 W cm-2) falling within the biological tissue transparency window, and the pronounced PL emission maxima in the visible and IR spectral regions minimize the effect of tissue autofluorescence and scattered exciting radiation on the registration of the target PL signal [14]. The combination of UCNPs with targeting agents specific to markers on the surface of tumor cells allows one to visualize even small tumor foci [15, 16].

Nevertheless, the effectiveness in the use of UCNPs in clinical practice is determined primarily by the absence or low level of toxicity to healthy cells of the brain. The risk of UCNPs internalization in normal cells and the development of toxic effects increases the chances of a loss of the functionally important links of neuron-glial networks, which can lead to neurological deficit and severe patient disability.

In the present work, UCNPs doped with the ytterbium and thulium ions (NaY0.794Yb_0.2_Tm_0.006_F_4_/NaYF_4_) were synthesized and characterized and their toxic effect on normal and tumor brain cells was studied.

## EXPERIMENTAL

UCNPs of the composition NaY0.794Yb_0.2_Tm_0.006_F_4_/NaYF_4_ were synthesized by solvothermal decomposition as described in [10]. Y_2_O_3_ (0.794 mM), Yb_2_O_3_ (0.2 mM), and Tm_2_O_3_ (0.006 mM) samples were placed in a threenecked flask, dissolved in 70% trifluoroacetic acid with heating and stirring, then cooled and evaporated. Sodium trifluoroacetate was added to the resulting precipitate to a final concentration of 2.2 mM; oleic acid and 1-octadecene (7.5 mL each) were then also added; the mixture was incubated at 100°C for 30 min with stirring in vacuum and at 180°C for 15 min under argon purge. The mixture was heated in Wood’s alloy at 343°C until the solution became cloudy, then incubated at 312°C for 25 min until a clear solution was obtained. The temperature was then reduced to 210°C, and 4 L of 1-octadecene was added. The resulting UCNPs crystals (core) were precipitated with isopropanol and centrifuged at 8 000 *g *for 12 min. The precipitate was resuspended in 3.5 mL of hexane and washed three times with 20 mL of ethanol.

A total of 60 mg of sodium trifluoroacetate, 140 mg of yttrium trifluoroacetate, 3 mL of oleic acid, and 3 mL of 1-octadecene were added to the UCNPs suspension; the mixture was placed in a three-necked flask and incubated at 100°C for 30 min with stirring in vacuum. The mixture was then incubated (for 15 min at 170°C) under argon purge and heated in Wood’s alloy to 290°C until the solution became cloudy and in the same conditions until the solution became transparent. The solution was cooled to 210°C, and 3 mL of 1-octadecene was added. The resulting core/shell UCNPs structures were precipitated, washed as described above, and re-suspended in chloroform.

**Analysis of the photophysical properties of UCNPs**

The photoluminescence emission spectrum of the UCNPs was obtained using a CM 2203 spectrofluorometer (SOLAR, Belarus) and an ATC-C4000-200AMF- 980-5-F200 semiconductor laser module with a wavelength of 978 nm (Semiconductor Devices, Russia).

**Measurement of the hydrodynamic diameter of UCNPs**

The hydrodynamic diameter of the UCNPs was measured by dynamic light scattering using a Zetasizer Nano ZS system (Malvern Instruments Ltd., UK) according to the manufacturer’s recommendations.

**Surface modification of UCNPs**

The UCNPs were coated with a polymer shell as described in [17]. A total of 130 μL of a solution of poly(- maleic anhydride-alt-1-octadecene) (PMAO) in chloroform (8 mg/mL) were added to 50 μL of the UCNPs suspension in chloroform (50 mg/mL). The mixture was sonicated for 30 s and incubated with stirring until 4/5 of the total volume had evaporated. The suspension was added dropwise to 1 mL of phosphate-buffered saline (PBS), stirred and sonicated for 30 min, until the chloroform had completely evaporated. The UCNPs were washed three times with PBS, then 40 μL of a poly(ethylene glycol) diglycidyl ether (PEG–GE) solution in deionized water (15 mg/mL) were added, mixed, and sonicated for 10 s. The mixture was incubated at 80°C for 30 min with periodic stirring and sonication. The resulting UCNP–PEG–DGE suspension was washed twice with PBS.

**Maintenance of glioma cell lines**

Murine glioma GL261 cells were cultured according to the cell culture data sheet in DMEM, supplemented with 4.5 g/L glucose (PanEco, Russia), 2 mM L-glutamine (PanEco), 0.11 g/L sodium pyruvate (Life Technologies, USA), and 10% fetal calf serum (PanEco). At the end of the exponential growth period, the cells were detached with a trypsin-versine solution (1 : 3) and reseeded. The multiplicity of seeding was 1 : 10, and the cell density was 1.0 × 10^5^ cells/mL.

Human glioma U-251 MG cells were cultured according to the cell culture data sheet in DMEM (PanEco) supplemented with 10% fetal calf serum (PanEco). At the end of the exponential growth period, the cells were detached with a trypsin-versine solution (1 : 3) and reseeded. The multiplicity of seeding was 1 : 5, and the cell density was 1.0 × 10^5^ cells/mL.

Experimental procedures with glioma cells were carried out after the third passage. Viability of the cell cultures was maintained in a Shel Lab CO_2_ incubator (Sheldon Manufacturing, USA) at a temperature of 35.5°C and a gas mixture containing 5% CO_2_.

**Isolation of primary hippocampal cultures**

Primary neuronal cultures were obtained from the embryonic hippocampal tissue of SHK mice (day 18 of gestation) in accordance with the protocol described in [18]. All work on experimental animals was carried out in accordance with the Rules for the Use of Experimental Animals (Russia, 2010) and International Guiding Principles (Code of Ethics) for Biomedical Research Involving Animals (CIOMS and ICLAS, 2012) with strict compliance with the ethical principles established by the European Convention for the Protection of Vertebrate Animals used for experimental and other scientific purposes (Strasbourg, 2006). Experimental animal studies were approved by the Bioethical Committee of the National Research Lobachevsky State University of Nizhny Novgorod. Pregnant females were euthanized by cervical vertebra dislocation. Next, embryos were removed from the uterus. The hippocampal tissue was subjected to mechanical and then enzymatic dissociation by 20-min incubation in a 0.25% trypsin solution (Life Technologies, USA). The obtained cell suspension was placed on coverslips (18 × 18 mm) pretreated with hydrophilic and positively charged polyethyleneimine (1 mg/mL, Sigma Aldrich, USA) to ensure effective attachment of the cells to the substrate. The initial cell density was 4,500 cells/cm2. Primary neuronal cultures were cultured in a Shel Lab CO_2_ incubator (Sheldon Manufacturing, USA) at 35.5°C and a gas mixture containing 5% CO_2_ for 21 days. The features of neuron-glial network formation were preliminarily evaluated using an Axio Observer A1 inverted fluorescence microscope (Carl Zeiss, Germany).

**Analysis of UCNPs toxicity against primary neuronal cultures and glioma cell lines**

Human glioma U-251 MG and murine glioma GL261 cells were seeded in a thin-bottom 48-well plate at 1.0 × 10^4^ cells per well. UCNPs were added to the culture medium at concentrations of 1, 10, 25, 50, and 100 μg/mL on the first day after the start of cultivation.

The cytotoxicity of UCNPs against tumor cells was evaluated by the MTT test 24 hours after the start of incubation [19, 20]. The tetrazolium dye 3-(4,5-dimethylthiazol- 2-yl)-2,5-diphenyltetrazolium bromide, which is reduced to a colored water-insoluble formazan by NADH-dependent dehydrogenases of viable cells, was added to the culture medium at a concentration of 0.5 mg/L. After a 60-min incubation, the culture medium was decanted and formazan crystals were dissolved in dimethyl sulfoxide (DMSO). The optical density of the solution was measured at 570 and 620 nm wavelengths using an Epoch microplate spectrophotometer (BioTek, USA). The number of viable cells (*N*v, %) was calculated using the following formula:

*N*_v_ = *E*_experimental_ / *E*_control_ × 100%.

On day 14 of cultivation of the primary hippocampal cultures, the UCNPs solution was added to the cultural medium at the same concentrations. Cell viability was assessed on days 3 and 7 after UCNPs addition according to [21], based on the ratio of dead cells stained with the fluorescent dye propidium iodide (Sigma Aldrich, USA) and the total number of cells stained with the fluorescent dye bisbenzimide (Sigma-Aldrich). Propidium iodide (5 μg/mL) and bisbenzimide (1 μg/mL) were added to the culture medium 30 min prior to the viability assessment. The cells were visualized using an Axio Observer A1 inverted fluorescence microscope (Carl Zeiss, 10×/0.2 Ph1 lens).

In addition, qualitative assessment of the cytotoxic effects was performed using the cytotoxicity scale (*Table*).

**Table:** Cytotoxicity score according to ISO 10993-5:2009

Cytotoxicity score	Number of dead cells in a culture, %	Cytotoxicity
0	0–10	Non-cytotoxic
1	10–20	Light
2	20–30	Average
3	> 30	Significant

**Analysis of the effect of UCNPs on the functional calcium metabolic activity of primary neuronal cultures**

The features of the functional calcium activity of the primary hippocampal cultures after UCNPs application were evaluated by calcium imaging. This method allows one to detect changes in the concentration of cytoplasmic Ca^2+^ ions, which are one of the key regulators of metabolic pathways in the cell, as well as to conduct a subtle analysis of the functional activity of both neurons and glia [22, 23]. Calcium events were detected using a specific calcium dye, Oregon Green 488 BAPTA-1 AM (OGB1) (Invitrogen, United States), and a Zeiss LSM 510 confocal laser scanning microscope (Carl Zeiss). OGB1 fluorescence was excited by an argon laser with λ = 488 nm and recorded using a 500- to 530-nm filter. Calcium events were analyzed using the Astroscanner software (certificate of state registration of a computer program No 2014662670). The following parameters were taken into account: duration of Ca^2^ oscillations (s), frequency of Ca^2+^ oscillations (number of calcium events/min), and number of cells exhibiting Ca^2+^ activity in the culture (%).

**Statistical data processing**

Data are presented as a mean ± standard error of the mean (SEM). The statistical significance of the differences between the experimental groups was determined using the ANOVA package in the SigmaPlot 11.0 software (Systat Software Inc.). Differences were considered statistically significant at *p* < 0.05.

## RESULTS AND DISCUSSION

**Synthesis and study of the properties of UCNPs**

**Fig. 1:**
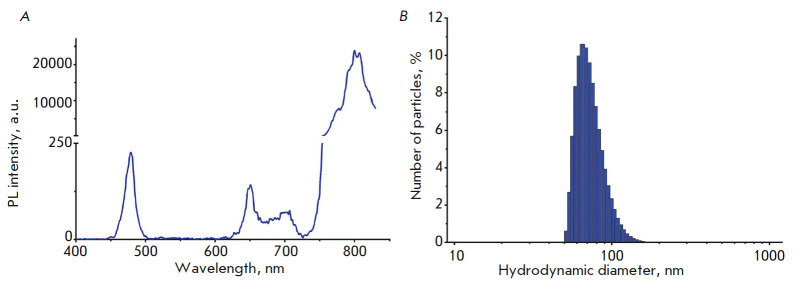
Properties of NaYF4:Yb,Tm UCNPs. *A *– PL emission spectra of the UCNPs excited by a 980-nm light; *B *– size distribution of the UCNPs with PMAO– and PEG–DGE-modified surface

Core/shell UCNPs of NaY0.794Yb_0.2_Tm_0.006_F_4_/NaYF_4_ composition were synthesized by solvothermal decomposition, coupled with thermal decomposition, which allowed for a transition from the cubic (α) phase to a more stable hexagonal (β) phase, which has a significantly higher coefficient of upconversion of brighter PL [11, 24, 25]. Trivalent lanthanide ions of ytterbium (Yb^3+^) and thulium (Tm^3+^) were used to dope the NaYF4 matrix, which allowed us to obtain UCNPs with PL emission maxima in the blue region (at a wavelength of 474 nm) and in the IR region (at a wavelength of 801 nm) with excitation at a wavelength of 980 nm (*Fig. 1A*). The presence of an inert NaYF4 shell increases the PL intensity of the UCNPs several-fold thanks to the absence of surface quenching effects in such core/shell structures [11]. An intense band in the IR spectral region (801 nm) falls within the biological tissue transparency window, which ensures the most photosensitive recording of the emission at this wavelength in an up to 1-cm-deep layer of a biological tissue [26, 27].

The obtained UCNPs of NaYF_4_:Yb,Tm composition carry hydrophobic oleic groups on their surface. The hydrophobic surface properties of the nanoparticles make them unstable in aqueous solutions and non-biocompatible. To stabilize and increase the biological compatibility of the UCNPs, they were hydrophilized by coating with an amphiphilic poly(maleic anhydride- alt-1-octadecene (PMAO). The hydrophobic chains of the PMAO molecules interact with the hydrophobic oleate residues, while the carboxyl groups of the PMAO remain exposed on the surface of the formed UCNPs coating, thus making them hydrophilic [10].

To further stabilize the structure of the UCNPs shell, poly(ethylene glycol) diglycidyl ether (PEG–DGE) was used. PEG–DGE epoxy groups interact with the carboxyl groups in PMAO, resulting in the integration of PEG–DGE in the structure of the UCNPs polymer shell, which increases their colloidal stability [17]. The hydrodynamic diameter of UCNPs coated with PMAO was 119 ± 9 nm, with a polydispersity index (PDI) of 0.662. After PEG–DGE addition, the average hydrodynamic diameter of the UCNPs decreased to 75 ± 15 nm with PDI = 0.136 (*Fig. 1B*), which indicates shell compaction and colloidal stabilization of the UCNPs.

Thus, the configuration and chemical composition of the UCNPs obtained allows one to allocate fluorescence excitation and emission maxima in the IR spectrum which corresponds to the biological tissue transparency window. This characteristic of the UCNPs will allow for a clear visualization of the target cells even in the deep layers of the brain. Recent studies on the photoluminescent characteristics of UCNPs with a similar chemical composition (NaYF4:Yb,Tm) revealed that laser scanning and wide-field fluorescence microscopy allows for high-resolution, high-contrast, and highspeed imaging of tumor cells with a high degree of specificity [28, 29].

Nevertheless, the key requirement for any fluorescent agent remains the absence or a minimum level of toxicity for non-target cells. For this reason, the next stage of our study was aimed at evaluating the cytotoxicity of the synthesized UCNPs against normal and tumor brain cells.

**Analysis of the UCNPs toxicity against U-251 MG and GL261 glioma cells**

**Fig. 2:**
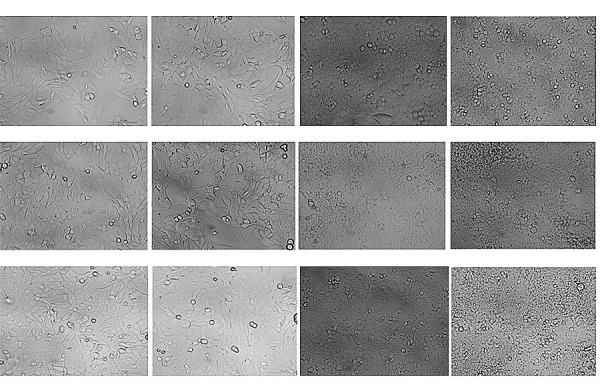
Representative wide-field light microscopy images of human U-251 MG and murine GL261 glioma cells after 24-hr incubation with the UCNPs

Analysis of UCNPs cytotoxicity against human (U-251 MG) and murine (GL261) glioma cells showed that the UCNPs, at the concentrations used, did not have a pronounced toxic effect on tumor cells (*Fig. 2*). The morphology and proliferation rates of the U-251 MG and GL261 cells were comparable with those for the control group. Actively dividing cells, as well as single cellular elements detached from the culture substrate and freely floating in the culture medium, which characterized one of the stages of monolayer formation, were observed in the view field.

**Fig. 3:**
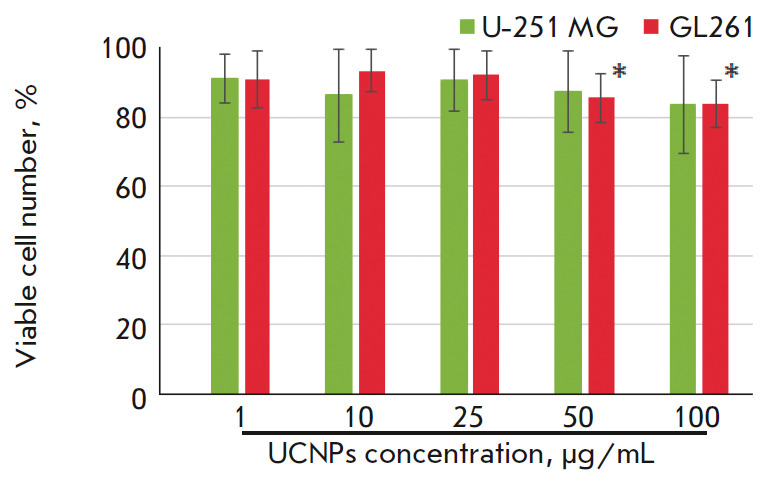
The effect of the UCNPs on the viability of human U-251 MG and murine GL261 glioma cells after 24-hr incubation with the UCNPs. The data represent a % of the control group; * – values relative to the control group were significant, *p * < 0.05, ANOVA

Evaluation of cell viability in the culture of human U-251 MG glioma cells by the MTT test did not reveal statistically significant differences from the control group (*Fig. 3*). A light cytotoxic effect on murine GL261 glioma cells was observed at UCNPs concentrations exceeding 50 μg/mL. The number of viable cells was 85.5 ± 7.1% for the “50 μg/mL UCNPs” group and 83.9 ± 7% for the “100 μg/mL UCNPs” group.

**Analysis of the UCNPs toxicity against primary hippocampal cultures**

The toxic effects of the UCNPs in normal brain cells were evaluated using primary hippocampal cultures. We had previously shown that primary neuronal cultures can serve as an adequate biological model of neuron-glial networks of the brain *in vitro *[30]. The toxicity analysis was performed on days 3 and 7 after UCNPs addition. Such a delayed cytotoxicity assessment was due to the metabolic features of normal brain cells, as well as longer degradation processes. Thus, in the presence of a toxic agent, the death of the major part of the cells occured within the first 3 days after exposure, while the cells that had lost a large number of connections and, thus, received an internal signal of programmed cell death died by day 7 [21].

**Fig. 4:**
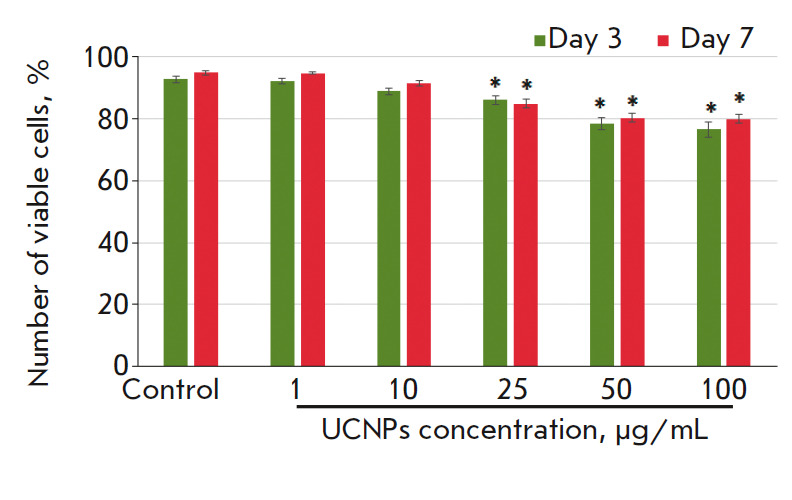
The effect of the UCNPs on the viability of primary hippocampal cultures within 7 days after incubation was started. * – values relative to the control group were significant, *p* < 0.05, ANOVA

The analysis showed that the UCNPs at concentrations of 1 and 10 μg/mL did not affect the viability of the primary hippocampal cells. On days 3 and 7 after the UCNPs addition, the number of dead cells in the experimental groups did not differ from that in the control. Manifestations of cytotoxic effects were noted in the groups of cell cultures treated with UCNPs at concentrations exceeding 25 μg/mL. On day 7 after UCNPs addition, the number of viable cells in the “25 μg/mL UCNPs” group was 84.9 ± 1.5%, which, according to the cytotoxicity scale, corresponds to a light cytotoxic effect. In the groups treated with the UCNPs at concentrations of 50 and 100 μg/mL, the number of viable cells in the primary cultures was 80.2 ± 1.4 and 79.9 ± 1.4%, respectively, which is deemed an average cytotoxic effect (*Fig. 4*).

Thus, the UCNPs did not have a pronounced cytotoxic effect on glioma cells. However, they caused a moderate cytotoxic effect in normal brain cells. A number of studies on the effect of various concentrations of UCNPs based on rare-earth elements have shown that these nanomaterials cause the death of 5% to 20% of the tumor cell population [31-33]. The low level of cytotoxicity is associated mainly with a high level of tumor cell metabolism, as well as their strong resistance to various pharmacological effects.

The brain tissue structure is characterized by a pronounced heterogeneity of the cellular composition. Neurons are most sensitive to the action of pharmacological and fluorescent agents. First of all, this is due to the low level of adaptive capabilities, which is largely because of the inferiority of the antioxidant enzymatic systems of this type of cells. Since brain neural networks are responsible for information processing, storage, and transmission, the death of 20% of functionally significant cells may be critical. The loss of elements of the neuron-glial networks will most likely lead to the complete elimination of neural network activity and, consequently, a dysfunction of the central nervous system.

For this reason, we further studied the effect of NaYF_4_:Yb,Tm UCNPs on the functional calcium activity of primary hippocampal cells.

**Analysis of the effect of the UCNPs on the functional activity of the neuron-glial networks of primary hippocampal cultures**

**Fig. 5:**
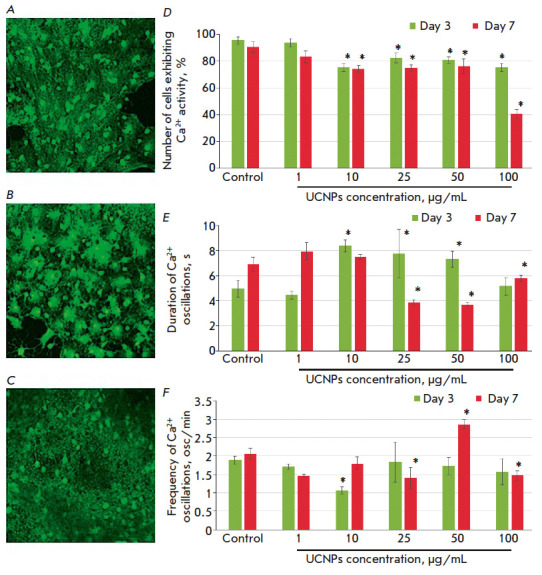
Features of the functional calcium activity of the cells in primary hippocampal cultures treated with the UCNPs. (*A*–*C*) Representative confocal images of primary hippocampal cultures stained with Oregon Green 488 BAPTA-1: *A *– control,* B *–10 μg/mL UCNPs, *C *–100 μg/mL UCNPs. (*D–F*) Main parameters of the spontaneous calcium activity in primary hippocampal cultures:* D *– percentage of cells exhibiting Ca^2+^ activity,* E *– duration of Ca^2+^ oscillations,* F *– frequency of Ca^2+^ oscillations

The study of the functional activity of primary hippocampal cells in the presence of the UCNPs by calcium imaging showed that high UCNPs concentrations cause significant changes in the spontaneous calcium activity of neuron-glial networks (*Fig. 5A-C*). A statistically significant decrease in the content (%) of the cells exhibiting Ca^2+^ activity was revealed on days 3 and 7 after the addition of the UCNPs at a concentration of 10 μg/mL and higher (*Fig. 5D*). The most pronounced effect was noted for the “100 μg/mL UCNPs” group, the content of working cells in which was 40.4 ± 3.4%, which is 2.2 times lower than that in the intact group.

Moreover, the use of high UCNPs concentrations led to a decrease in the duration of calcium oscillations. By the 7^th^ day of the experiment, this parameter value was 3.9 ± 0.2, 3.7 ± 0.2, and 5.8 ± 0.3 s for the groups treated with the UCNPs at concentrations of 25, 50, and 100 μg/mL, respectively, which significantly differed from the values in the intact group (*Fig. 5E*). The frequency of Ca^2+^ oscillations was also significantly altered for UCNPs at a concentration of 10 μg/mL or higher. The UCNPs at a concentration of 100 μg/mL contributed to a statistically significant decrease in the frequency of calcium oscillations, which was most likely due to the death of a large number of functionally active cells in the culture.

Thus, the UCNPs at concentrations of 25, 50, and 100 μg/mL had an average toxic effect on primary hippocampal cells, which was manifested in a decreased viability and significant changes in the functional activity of neuron-glial networks. It was previously shown that the toxic properties of nanoparticles are largely determined by the chemical composition of the shell [34]. In this regard, the development of new types of UCNPs coatings contributing to a decrease in toxicity for normal brain cells and an increase in the effectiveness of a theranostic agent against tumor cells seems promising. The creation of multimodal hybrid structures based on UCNPs [35] significantly expands the possibilities of their use for onco-theranostics.

## CONCLUSION

The physicochemical properties of UCNPs doped with the ytterbium and thulium ions (NaY_0.794_Yb_0.2_Tm_0.006_F4/NaYF_4_) allow one to use them as agents for deep optical imaging of tumor cells in the brain. The UCNPs were shown to have no cytotoxic effect on tumor cells (glioma cells). However, at high concentrations, they exert a cytotoxic effect in primary neuronal cultures, which is characterized by a decrease in cell viability and changes in the functional activity of neuron-glial networks. Thus, the use of UCNPs as fluorescent markers necessitates a study of the rate of accumulation and excretion of the UCNPs in tumor and healthy brain cells, as well as the possibility of modifying the surface of nanoparticles in order to reduce the toxic effects.

## Abbreviations

BBB: blood–brain barrier
DIV: day of culture development in vitro
DMSO: dimethyl sulfoxide
IR: infrared
PBS: phosphate-buffered saline
PDI: polydispersity index
PEG–DGE: poly(ethylene glycol) diglycidyl ether
PL: photoluminescence
PMAO: poly(maleic anhydride-alt-1-octadecene)
UCNPs: upconversion nanoparticles
